# Assessment and Management of Atopic Dermatitis in Primary Care Settings: A Systematic Review

**DOI:** 10.7759/cureus.44560

**Published:** 2023-09-02

**Authors:** Saad M Alqahtani, Bassam H Awaji, Abdulaziz M Mahdi, Fatimah H Althawab, Hadeel M Aljohani, Raghad A Rayes, Rahaf K Shafie, Raneem Abdulrahman Aljohani, Sarah Alkhorayef, Mohammed K Alghamdi

**Affiliations:** 1 Family Medicine, King Salman Armed Forces Hospital, Tabuk, SAU; 2 Faculty of Medicine, University of Tabuk, Tabuk, SAU; 3 Orthopedic Surgery, University of Jeddah, Jeddah, SAU; 4 Medicine and Surgery, King Faisal University, Hofuf, SAU; 5 Medicine and Surgery, King Abdulaziz University Faculty of Medicine, Jeddah, SAU; 6 Family Medicine, King Abdulaziz University Faculty of Medicine, Jeddah, SAU; 7 Medicine, Ibn Sina National College for Medical Studies, Jeddah, SAU; 8 Dermatology, Faculty of Medicine, University of Tabuk, Tabuk, SAU; 9 Medicine and Surgery, Ibn Sina National College for Medical Studies, Jeddah, SAU; 10 Medicine, University of Jeddah, Jeddah, SAU

**Keywords:** inflammatory skin condition, early childhood, eczema, primary care provider, atopic dermatitis

## Abstract

Atopic dermatitis is a complex, recurrent, chronic inflammatory skin condition. It frequently begins to manifest in early childhood and may last throughout adulthood. The need for clinical practice guidelines that are based on evidence is critical for efficient and secure care. Little is known about how primary care providers (PCPs) should handle pediatric and adult atopic dermatitis cases and whether they should follow national recommendations. Our systemic review aimed to examine management strategies for treating adult and pediatric (family) atopic dermatitis, including topical calcineurin inhibitors (TCIs), topical corticosteroids (TCS), skin emollients, oral antihistamines, and diet. Data sources were PubMed (MEDLINE) and Embase. Our review investigated English-language articles from 2014 to 2023 that studied the management of adult and children atopic dermatitis. Overall, there were 15 articles included. Surveys and analyses of national databases were the most widely used methods (n=7). The use of TCS by PCPs was common, but they also overprescribed nonsedating antihistamines, favored low-potency drugs, and avoided TCIs. Most studies relied on healthcare personnel reporting their typical behaviors rather than looking at specific patient encounters and it is considered a limitation. Finally, there are gaps in knowledge and management of critical topics such as prescribing TCIs and understanding the safety profiles of TCS, when it comes to treating adult and pediatric atopic dermatitis. Future research in this area is urgently needed because the current systemic assessment is mostly restricted to small studies that assess prescribing behaviors with scant information describing nonmedication management.

## Introduction and background

Atopic dermatitis (AD), also known as atopic eczema, is a chronic, itchy, recurrent, systemic, inflammatory skin condition that is frequently associated with other atopic systemic conditions such as asthma, allergic rhinitis, and food allergies. Globally, most instances of AD are discovered before the age of five years. The key to preventing AD problems and raising the quality of life (QoL) is early diagnosis and prompt treatment. Around 15-20% of children and 2-10% of young people are affected by AD, which is most common in this age group. In emerging nations, such as those in Africa and the Middle East, the prevalence of AD has been seen to be rising [1].

Complex environmental, immunological, and genetic risk factors are all part of the pathogenesis of AD. Inflammatory conditions in the cutaneous region and structural and functional issues in the epidermal region are the disease's two primary pathophysiological characteristics, and both are brought on by insufficient immune responses mounted against antigens that have invaded the skin. Furthermore, AD lesions include more Th2 cells, which are the sources of localized inflammation [2]. Increased epidermal thickness with sprouting nerve fibers, increased immunostimulatory chemokine production, and significant infiltration are the main pathophysiologic characteristics of AD lesions [3]. It was once thought to be a childhood illness caused by an imbalance in the T-helper-2 response and overactive IgE reactions to allergens, but today it is understood to be a lifelong disposition with a variety of clinical symptoms and expressivity, with abnormalities of the epidermal barrier playing a key role. The goal of current prevention and therapy is to restore the function of the epidermal barrier, which can best be done by using emollients. In addition to being used proactively in conjunction with topical calcineurin inhibitors to maintain remission, topical corticosteroids are still the first-line treatment for acute flares. Although tailored disease-modifying therapies are being developed, severe refractory patients are being treated with non-specific immunosuppressive medications [4].

AD is characterized by debilitating signs and symptoms, in addition to other illnesses including food allergies, allergic rhinitis, allergic conjunctivitis, asthma, and viral, bacterial, and fungal skin infections. Xerosis (dryness), diffuse erythematous patches, excoriated, leaky papulovesicular, and lichenified plaques with chronic lesions, itching, and pain are some of the clinical indications of AD [5]. Due to symptoms including frequent sleep interruptions, discomfort, pain, melancholy, anxiety, and limitations in daily tasks like mobility and self-care, severe AD hurts a patient's quality of life (QoL).

To control and treat AD, all facets of the disease, including clinical symptoms and patient QoL, should be addressed. Given the significant prevalence of AD in children around the world, primary care providers (PCPs), such as pediatricians and general practitioners, are frequently responsible for managing the condition. These professionals report receiving insufficient training on AD during residency. While controlling asthma, a prevalent atopic condition with various therapeutic facets, is the subject of a substantial literature review, little is known about how PCPs treat juvenile AD [6]. Therefore, the purpose of this systematic review is to investigate how PCPs handle the many aspects of managing AD in children and adults, including prescription, counseling, referring for ancillary testing, and disease understanding. We also looked for distinctions between PCPs' and dermatologists' approaches to treating adult and pediatric AD.

## Review

Methods

The procedures for the current systemic review were developed according to the Preferred Reporting Items for Systematic Reviews and Meta-Analyses (PRISMA) standards. Keywords and Medical Subjects Headings (MeSH) phrases were used to search PubMed (MEDLINE), Embase, and Cochrane databases for articles published in the English language from 2014 to August 2023 on AD, PCP involvement in AD, and management components like counseling, medication, and auxiliary testing.

**Figure 1:**
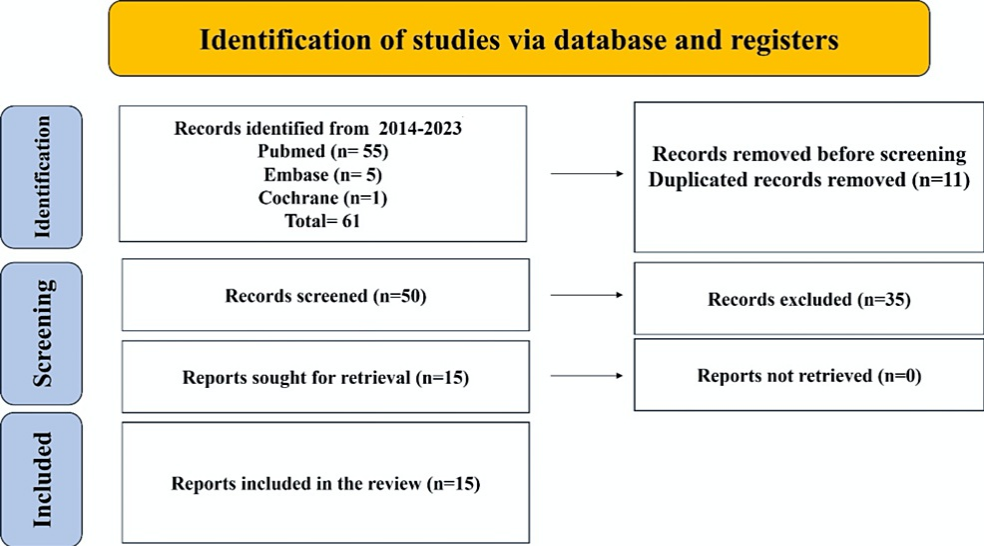
Study flow diagram

Examining the references found in the identified papers led to additional studies. English articles and reviews demonstrating possible management of atopic dermatitis in families (adults and children) with an age range of 0-65 years old, in which family background about how to deal with the disease was given, were included. Articles with incomplete data on the management of AD and those published before 2014 were excluded. After retrieving all available full texts and excluding all that did not fit our inclusion criteria, 15 articles were included as part of this review. Figure 1 shows the process used for search screening and article shortlisting.

Results

Table 1 provides a description of some included studies.

**Table 1:** Description of a few included studies AD: atopic dermatitis; PCP: primary care provider; TCI: topical calcineurin inhibitors; TCS: topical corticosteroids; JAK: Janus kinase; TEAE: treatment-emergent adverse event

Serial no.	Author and year	Aim of the study	Conclusion
1	Frazier and Bhardwaj.(2020) [7]	Emollients are liberally applied as part of maintenance therapy, and regular baths with cleaner without soap are recommended. The first line of defense against flare-ups of AD is the use of topical corticosteroids. Topical calcineurin inhibitors like pimecrolimus and tacrolimus are first-line treatments that work best when combined with topical corticosteroids. When first-line treatments are ineffective, ultraviolet phototherapy is a safe and efficient treatment for moderate to severe AD. Antibiotics against staphylococci are useful in treating secondary skin infections. Since oral antihistamines do not lessen pruritus, they are not advised. There is insufficient research to back up the use of integrative medicine to treat AD.	Although newer drugs like crisaborole and dupilumab that have been licensed by the FDA are efficient at treating AD, the cost of these drugs makes them unaffordable for most patients right now.
2	Young et al. (2021) [1]	Management of pediatric AD by PCPs, including TCIs, TCS, antihistamines, bathing, emollients, and nutrition.	When it comes to treating pediatric AD, PCPs have management and knowledge gaps, especially when it comes to prescribing TCIs and comprehending TCS safety profiles. Future investigations in this field are critically needed because the existing literature is largely made up of small studies that evaluate prescribing practices and provide few details on non-medication management.
3	Torres et al. (2019) [8]	Topical anti-inflammatory medications are to be considered for chronic or recurrent lesions or illness flare-ups. If there is a poor response, systemic immunosuppressants, phototherapy, and more recently, dupilumab, should be administered. However, treating moderate-to-severe atopic dermatitis is still difficult, and we urgently need new, effective, safe, and tailored therapies.	Future research in AD will continue investigating gene-environment interactions and how they influence pathophysiology, disease severity, and treatment outcomes, even though the last few years have seen significant improvements in our understanding of the condition.
4	Strathie et al. (2016) [9]	Comprehensive overview of how AD is treated in pediatric general practice settings, and the evidence for management, the link to allergies, and when it is important to refer to specialists.	It takes a detailed grasp of AD to prescribe topical steroids to young children who have it. To allay parents' fears over the long-term negative effects of TCS, it is important to adequately explain the treatment to them. The contact between the doctor, families, and kids will be more fulfilling if general practitioners are aware and at ease discussing AD.
5	Chovatiya and Paller. (2021) [10]	Japan just approved delgocitinib ointment for both pediatric and adult AD. Oral JAKi include baricitinib (JAK1/2), abrocitinib (JAK1), and upadacitinib (JAK1). All three met the primary and secondary goals of numerous trials for moderate-to-severe AD. The mild-to-moderate TEAEs included acne, nausea, headaches, upper respiratory tract infections, and to a lesser extent, herpes infection and certain test abnormalities. JAKi has a great deal of potential as the subsequent phase of targeted AD therapy.	Even though their remarkable efficacy is offset by a favorable safety profile in clinical research, real-world data are necessary to better understand long-term safety, durability, and therapeutic efficacy.
6	Nakashima et al. (2021) [11]	The discovery of novel medications is being driven by our growing understanding of AD's etiology.	Each medication produced notable symptom improvements with a low frequency of side effects. Oral JAK inhibitors have the power to rapidly alleviate pruritus and skin complaints. Considering this, the development of these topical and oral JAK inhibitors would be seen as a breakthrough in the management of AD.
7	Salvati et al. (2021) [12]	Age, ethnicity, gender, the morphology and location of skin lesions, the severity of the disease in terms of the body surface area affected and the intensity of symptoms (especially pruritus), the duration of the disease, the frequency of AD relapses, the response to previous AD treatments, coexisting conditions (such as mucosal atopy, immunodeficiency, etc.), potential concomitant therapies, and the impact on the patient's quality of life are the main factors, The practical application of proven AD-specific biomarkers in the selection/stratification and monitoring of AD patients will represent the future potential of personalized therapy choices in AD.	Both conventional and novel therapies for AD focus on key biological pathways involved in the disease's etiology. The arsenal against AD has recently increased quickly and favorably thanks to biological therapies and JAK inhibitors, thus the end of the dry spell does not appear so far away.

Discussion

This review highlights AD in children and adults to find more appropriate treatment options. The treatment of AD in adults and children is complicated and necessitates both medication and lifestyle changes. Fifteen studies focusing on how PCPs manage adult and pediatric AD across several domains were found in our systematic review.

AD can manifest differently in adults than children. Adults have more signs of chronic disease, higher prevalence, different patterns of hand eczema, and a stronger relationship of disease activity with emotional factors, whereas children with AD have more exudative lesions, perifollicular accentuation, pityriasis alba, Dennie-Morgan folds, and seborrheic dermatitis-like presentation [13]. Diagnosis is mainly based on physical examination and symptoms.

The first line of defense against flare-ups of atopic dermatitis is the application of topical corticosteroids (TCS) [7]. Tacrolimus and pimecrolimus are topical calcineurin inhibitors that can be utilized as first-line treatments in conjunction with TCS [12]. The regular use of TCS (with a preference for low-potency TCS and avoidance of higher potency alternatives), avoidance of topical calcineurin inhibitors (TCI), and overprescribing of nonsedating antihistamines by PCPs were some of the themes that emerged in relation to medication management. TCS is a fundamental component of pediatric AD management, where they are applied on individuals who do not have disease-free intervals with simple skincare and emollients alone to treat and/or prevent flares. While many healthcare professionals are familiar with topical hydrocortisone (a class VII TCS), medium-potency TCS is safe with proper monitoring and can be justified in the primary care setting for AD involving specific body regions outside of the face [14].

Even though studies by Rea et al. [15], Craddock et al. [16], and Fleischer [17] indicate that PCPs frequently prescribe and/or recommend TCS to kids with AD, it is seen that PCPs generally showed a preference for lower potency TCS, such as hydrocortisone [18]. Importantly, mild TCS like 1% hydrocortisone can be obtained over the counter and are not included in analyses of prescribing databases, indicating that the percentage of PCPs who recommend hydrocortisone for pediatric AD may be larger than the percentages identified in our literature search. Concern for side effects and TCS phobia likely play important roles in PCPs’ preference for low-potency agents [19]. The cornerstone of anti-inflammatory therapy is topical corticosteroids (TCS), which are utilized in the management of AD in both adults and children. They affect several immune cells, including T lymphocytes, monocytes, macrophages, and dendritic cells, impairing the processing of antigens, and stifling the release of cytokines that promote inflammation. When lesions do not improve with frequent moisturizing and appropriate skin care, they are often included in the treatment plan [20].

Managing AD in children requires, in part, giving parents a thorough explanation to allay their worries about the potential side effects of TCS. The contact between the doctor, families, and kids will be more fulfilling if general practitioners are aware of and at ease with AD [9]. Up to 83% of parents are apprehensive about TCS, and this can negatively impact treatment outcomes. The relatively high levels of TCS phobia found among PCPs in our review may be a factor in parental TCS phobia [21]. TCIs, in contrast to TCS, were not frequently recommended by PCPs due to worries about lymphoma adverse effects; however, this danger is purely hypothetical, and numerous studies have been unable to show a link between long-term usage of TCIs and a risk of malignancy [22].

A new and advanced treatment strategy for the management of AD is using topically applied and systemically administered Janus kinase (JAK) inhibitors (JAKi) such as topical ruxolitinib (JAK1/2) and delgocitinib while baricitinib (JAK1/2), abrocitinib (JAK1), and upadacitinib (JAK1) are oral treatment strategies for mild-to-moderate AD. Ruxolitinib (JAK1/2) and delgocitinib (pan-JAK) are two new topical JAK inhibitors. In phase 3 clinical studies for mild-to-moderate AD, roxolitinib cream achieved all primary and secondary endpoints with a low incidence of treatment-emergent adverse events (TEAEs). Recently, delgocitinib ointment for both pediatric and adult AD received Japanese approval. The primary and secondary objectives of multiple trials for moderate-to-severe AD were satisfied by all three. Acne, nausea, headaches, upper respiratory tract infections, and to a lesser extent, herpes infection, and certain test abnormalities were among the mild-to-moderate TEAEs. As the next generation of targeted AD therapy, JAKi holds a lot of potential. Real-world data are required to better understand long-term safety, even though their exceptional efficacy is tempered by a favorable safety profile in clinical studies [10].

We discovered that PCPs frequently prescribe oral antihistamines for AD, that sedating antihistamines may be helpful for kids with pruritus that keeps them awake, and that nonsedating antihistamines can be useful in controlling comorbid illnesses including allergic rhinitis. Nonsedating antihistamines, on the other hand, have no place in the treatment of AD, and there is some debate as to whether using them can even be harmful, as children with AD who take antihistamines may experience worsening symptoms of hyperactivity and inattention [23].

Ultraviolet phototherapy appears to be a safe and effective treatment for moderate to severe AD when first-line treatments are not adequate. Antistaphylococcal antibiotics are effective in treating secondary skin infections. Oral antihistamines are not recommended because they do not reduce pruritus. Evidence is lacking to support the use of integrative medicine in the treatment of atopic dermatitis. Newer medications approved by the United States Food and Drug Administration such as crisaborole and dupilumab are effective in treating AD but are currently cost-prohibitive for most patients [7].

Emollients are liberally applied as part of maintenance therapy, and regular baths without soap are recommended [12]. Richer moisturizing products like lotions and ointments are preferred. Regular application of emollients reduces symptoms and lowers the requirement for topical anti-inflammatory drugs. Studies that looked at emollient management discovered that while not always, PCPs frequently advise children with AD to take emollients [24].

The evidence regarding the effectiveness of exercise therapy in the management of AD is diverse [1]. We only found a small amount of information on PCP management of food and allergy in AD. Dietary restrictions may not only be ineffective but also result in poorer nutrition, increased parental anxiety, and a higher risk of developing food allergies because they reduce children's chances of developing early tolerance to certain foods, such as peanuts [25].

## Conclusions

Our review was limited by the facts that many of the included studies used questionnaires to assess general provider behaviors rather than concentrating on patient experiences and the number of samples was frequently small. The severity of the patient's sickness, the healthcare system they work in, and local rules all differ, and as AD is a dynamic inflammatory disease, it has several treatment options starting from topical corticosteroids, topical and oral JAK inhibitors, oral anti-histaminic and finally some dietary restrictions.
